# Effects of autophagy on apoptosis of articular chondrocytes in adjuvant arthritis rats

**DOI:** 10.1111/jcmm.14629

**Published:** 2019-09-11

**Authors:** Renpeng Zhou, Fei Zhu, Xiaoshan Wu, Sujing Song, Yong Chen, Chuanjun Zhu, Beibei Dai, Xuewen Qian, Ke Wang, Wei Hu, Feihu Chen

**Affiliations:** ^1^ Department of Clinical Pharmacology The Second Hospital of Anhui Medical University Hefei China; ^2^ Anhui Key Laboratory of Bioactivity of Natural Products, School of Pharmacy Anhui Medical University Hefei China; ^3^ The Key Laboratory of Anti‐inflammatory and Immune Medicine, Ministry of Education Anhui Medical University Hefei China

**Keywords:** 3‐methyladenine, apoptosis, autophagy, chondrocyte, rapamycin, rheumatoid arthritis

## Abstract

Rheumatoid arthritis (RA) is a chronic, systemic autoimmune disease that eventually leads to joint deformities and loss of joint function. Previous studies have demonstrated a close relationship between autophagy and the development of RA. Although autophagy and apoptosis are two different forms of programmed death, the relationship between them in relation to RA remains unclear. In this study, we explored the effect of autophagy on apoptosis of articular chondrocytes in vivo and in vitro. Adjuvant arthritis (AA) and acid‐induced primary articular chondrocyte apoptosis were used as in vivo and in vitro models, respectively. Articular chondrocyte autophagy and apoptosis were both observed dynamically in AA rat articular cartilage at different stages (15 days, 25 days and 35 days). Moreover, chondrocyte apoptosis and articular cartilage injury in AA rats were increased by the autophagy inhibitor 3‐methyladenine (3‐MA) and decreased by the autophagy activator rapamycin. In addition, pre‐treatment with 3‐MA increased acid‐induced chondrocyte apoptosis, while pre‐treatment with rapamycin reduced acid‐induced chondrocyte apoptosis in vitro. These results suggest that autophagy might be a potential target for the treatment of RA.

## 1 INTRODUCTION

Rheumatoid arthritis (RA) is a chronic, systemic autoimmune disease characterized by synovial hyperplasia, immune cell infiltration, and cartilage and bone destruction.^1^ Recently, articular chondrocyte death has been regarded as one of the main causes in the destruction and loss of articular cartilage of RA.^2^ Increasing evidence suggests that autophagy and apoptosis are closely associated with the occurrence and development of RA.^3^ However, the relationship between autophagy and apoptosis in RA remains unclear. It is therefore necessary to determine the underlying roles of autophagy and apoptosis in articular cartilage destruction.

Autophagy appears to be important for maintaining basal cellular homeostasis under normal conditions and also plays a crucial pro‐survival role in cell homeostasis under stress conditions, such as starvation or stress due to growth‐factor deprivation.^4^ However, consequent excessive autophagy may lead to autophagic cell death (type II programmed cell death), which differs from apoptosis and necrosis.^5^ Apoptosis can be induced via either an intrinsic or an extrinsic pathway.^6^ Apoptotic changes in cell morphology include cell shrinkage, nuclear chromatin margination and the formation of apoptotic bodies.^7^ Such information on apoptosis furthers our understanding of the pathophysiological of RA and supports the identification of novel therapeutic targets. The relationship between autophagy and apoptosis is complex and is critical in terms of the ultimate fate of the cell.^8^ Autophagy and apoptosis thus jointly regulate cell survival and death; however, their relationship in terms of RA chondrocytes is still unclear.

In this study, we examined the dynamic changes in autophagy and apoptosis in adjuvant arthritis (AA) rats at different stages. We also evaluated the effects of inhibiting or inducing autophagy using 3‐methyladenine (3‐MA) or rapamycin (Rapa), respectively, on the expression of apoptosis markers in AA rat articular cartilage in vivo, and detected the effect of autophagy on acid‐induced articular chondrocyte apoptosis in vitro.

## 2 MATERIALS AND METHODS

### 2.1 Animals and drug application

140‐160 g male Sprague Dawley rats were obtained from the Experimental Animal Center of Anhui Medical University. All animal experimental procedures were approved by the Animal Care and Use Committee of Anhui Medical University.

The rats were randomly divided into four groups (n = 8 each): normal group and 15‐, 25‐ and 35‐day AA groups. Second, a further 32 rats were also divided into four groups (n = 8 each): normal group; AA group; 3‐MA‐treated AA group; and triamcinolone acetonide (TA)‐treated AA group. Third, another 32 rats were divided into the following four groups: normal group; AA group; Rapa‐treated AA group; and TA‐treated AA group. AA was induced by intraplantar injection of 0.1 mL Complete Freund's Adjuvant (CFA; Sigma). 3‐MA (15 mg/kg/d) and Rapa (2 mg/kg/d) (Sigma‐Aldrich) were administered by intraperitoneal injection from days 14‐24. TA (1 mg/kg, once every 3 days for three times) (Kunming Jida Pharmaceutical Co., Ltd) was administered by intra‐articular injection. Next, the rats were anesthetized and killed, and serum, cartilaginous tissues and ankle joints were collected for further study.

### 2.2 Histology and immunohistochemistry

Ankle joints were harvested and fixed in 4% paraformaldehyde for 48 hours, decalcified with 10% EDTA solution, embedded in paraffin and sectioned longitudinally (4 µm) from the medial compartment of the joint. Serial paraffin sections were stained with haematoxylin and eosin (H&E) and analysed for joint pathological changes. Briefly, immunohistochemical sections were incubated with primary anti‐rat collagen type II antibody (diluted 1:200) or LC3 antibody (diluted 1:200) overnight at 4°C, followed by secondary goat antimouse IgG (diluted 1:200) for 30 minutes the following day. Positive nuclei were stained brown.

### 2.3 TUNEL and LC3 immunofluorescence double staining

For terminal deoxynucleotidyl transferase dUTP nick end labelling (TUNEL) analysis, sections were stained by TUNEL and LC3 immunofluorescence double staining according to the manufacturer's instructions (Roche). Finally, images were viewed under a fluorescence microscope. Apoptotic cells appeared green, and cells labelled by LC3 primary antibody were red.

### 2.4 Articular chondrocyte isolation and culture

Primary rat articular chondrocytes were extracted from male Sprague Dawley rats (weighing 140‐160 g), as described previously.^9^

### 2.5 Apoptosis analysis

After different treatments, the chondrocytes were washed with PBS and then stained with Hoechst 33342 according to the manufacturer's instructions for 15 minutes in the dark. After staining, the cell apoptotic features were observed and photographed under an inverted fluorescence microscope (Olympus). Cell apoptosis rates were measured using an Annexin V‐FITC kit (Bestbio). Stained cells were measured by flow cytometry (BD Biosciences), and apoptosis rates were analysed using FlowJo software (Tree Star).

### 2.6 Western blotting

We examined the expression of LC3, Beclin‐1, caspase‐3, caspase‐9, PARP (antibodies diluted 1:1000, Cell Signaling Technology), as well as type II collagen, and β‐actin (antibodies diluted 1:500) by Western blotting assay as described previously.^2^

### 2.7 Statistical analysis

All the data were expressed as mean ± standard deviation. Means were compared among groups by one‐way analysis of variance (ANOVA). A value of *P* < .05 was considered to be statistically significant.

## 3 RESULTS

### 3.1 Dynamic changes in autophagy and apoptosis in articular chondrocytes in AA rat model

**Figure 1:**
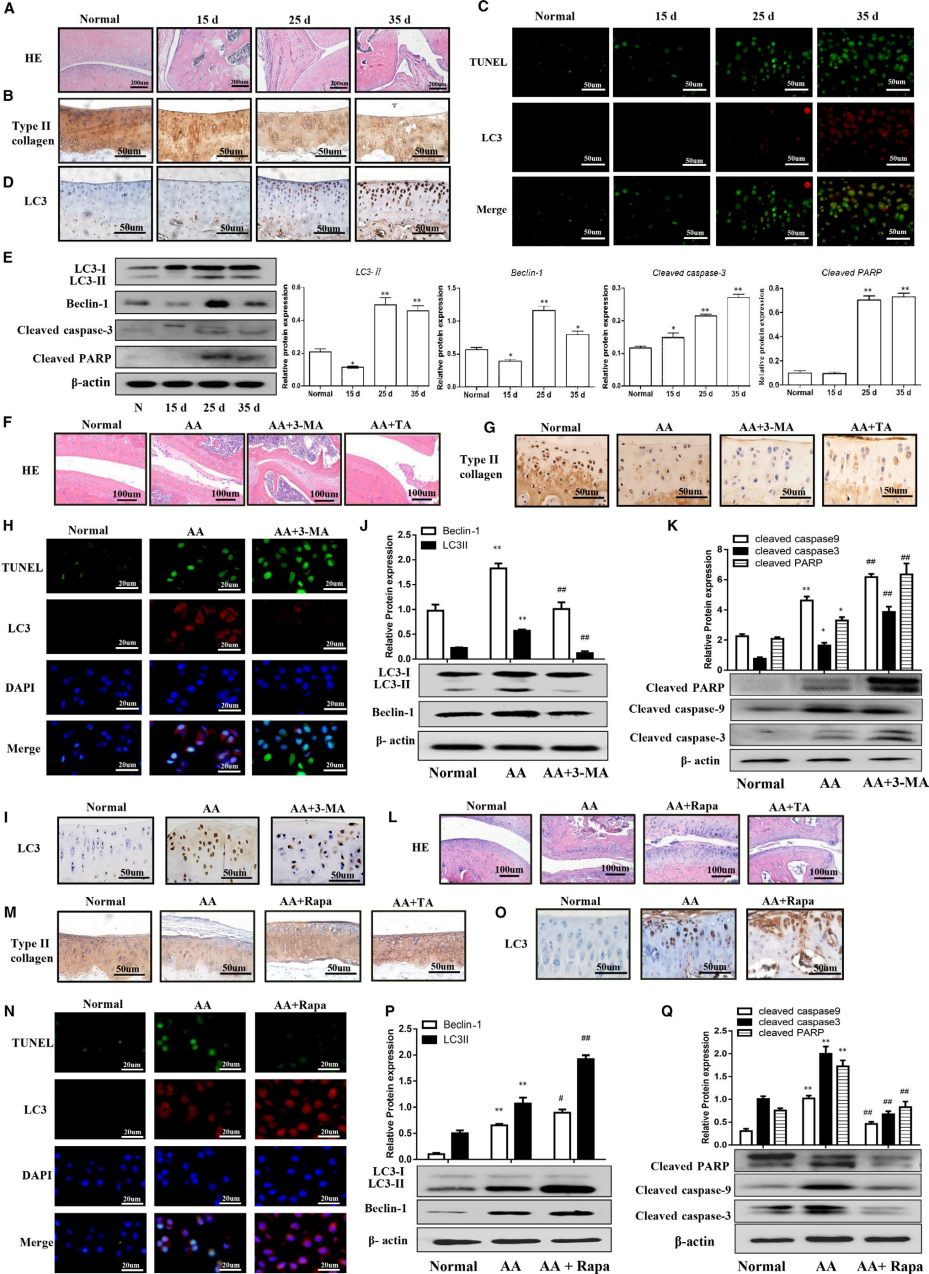
Effects of autophagy on apoptosis of AA rat articular chondrocytes in vivo. A, H&E staining for cartilage tissues of AA rats at different stages. B, Immunohistochemical staining for type II collagen. C, The co‐expression of autophagy‐related and apoptosis‐related markers were assessed by TUNEL and immunofluorescence double staining. D, Immunohistochemical staining for LC3. E, Autophagy and apoptosis protein expressions were detected by Western blotting. *P < .05 and **P < .01 vs normal group. F, G, H&E staining and Immunohistochemical staining. H, The co‐expression of autophagy‐related and apoptosis‐related markers were assessed by TUNEL and immunofluorescence double staining. I, Immunohistochemical staining for LC3. J, Autophagy protein expressions were detected by Western blotting. K, Protein expressions of apoptosis were detected by Western blotting. *P < .05 and **P < .01 versus normal group, ^##^ *P* < .01 vs AA group. H&E staining for cartilage tissues in different treatment groups (L), immunohistochemical staining for type II collagen (M), TUNEL and immunofluorescence double staining (N), immunohistochemical staining for LC3 (O), Western blotting for autophagy protein expressions (P) and apoptosis protein expressions (Q). *P < .05 and **P < .01 vs normal group, ^##^ *P* < .01 vs AA group

H&E staining showed that the articular cartilage destruction, synovial hyperplasia and inflammation in AA rats (Figure 1A). Moreover, immunohistochemical staining demonstrated that type Ⅱ collagen gradually decreased, while LC3 expression was increased with prolonged AA modelling time in articular cartilage (Figure 1B and 1D). We also examined autophagy and apoptosis in chondrocytes by detecting apoptotic cells using TUNEL (green) and autophagy by LC3 immunofluorescence (red). Low TUNEL and LC3 signals were observed in normal cartilage, while both were increased in cartilage of AA (Figure 1C). Western blotting also showed that LC3‐II and Beclin‐1 protein expression in articular cartilage were slight decreased at 15 days after CFA injection, but were significantly increased at 25 and 35 days compared with the normal group. Moreover, cleaved caspase‐3 and cleaved PARP were increased after CFA injection in time‐dependent manners (Figure 1E). These results indicated that articular chondrocytes undergo cell death during the progression of arthritis via a combination of autophagy and apoptosis.

### 3.2 Inhibition of autophagy by 3‐MA enhanced articular chondrocyte apoptosis in AA rats

We investigated the effect of autophagy on chondrocyte apoptosis by inhibiting autophagy using 3‐MA. 3‐MA treatment enhanced joint and cartilage damage compared with the AA group, as shown in Figure 1F. Consistently, immunohistochemistry also showed a decrease in type II collagen in 3‐MA‐treated AA rats (Figure 1G). In addition, TUNEL and LC3 immunofluorescence double staining showed that 3‐MA also decreased LC3‐positive staining and increased TUNEL‐positive staining in AA rat articular cartilage (Figure 1H). Moreover, LC3‐positive cells were reduced after 3‐MA treatment (Figure 1I) and 3‐MA could inhibit LC3‐II and Beclin‐1 expression (Figure 1J), while significantly increased expression of cleaved PARP, cleaved caspase‐3 and cleaved caspase‐9 in articular cartilage compared with the AA group (Figure 1K). Taken together, these results suggested that inhibition of autophagy by 3‐MA could enhance articular chondrocyte apoptosis in an AA rat model.

### 3.3 Activation of autophagy by Rapa reduced articular chondrocyte apoptosis in AA rats

We further investigated the effect of autophagy activation using Rapa on apoptosis of articular chondrocytes in AA rats. As shown in Figure 1L, Rapa reduced joint and cartilage damage compared with the AA group. Moreover, type II collagen was shown to be increased (Figure 1M), and TUNEL and LC3 immunofluorescence double staining revealed that Rapa increased LC3‐positive staining and reduced TUNEL‐positive staining in AA rat articular cartilage (Figure 1N). Furthermore, LC3‐positive staining was increased in Rapa‐treated AA rats (Figure 1O) and Western blotting demonstrated that Rapa up‐regulated LC3‐II and Beclin‐1, decreased cleaved PARP, cleaved caspase‐3 and cleaved caspase‐9 in articular cartilage compared with the AA group (Figure 1P and Q). Taken together, these results indicated that activation of autophagy by Rapa could attenuate articular chondrocyte apoptosis in an AA rat model.

### 3.4 Inhibition of autophagy by 3‐MA enhanced apoptosis in primary rat articular chondrocytes

**Figure 2:**
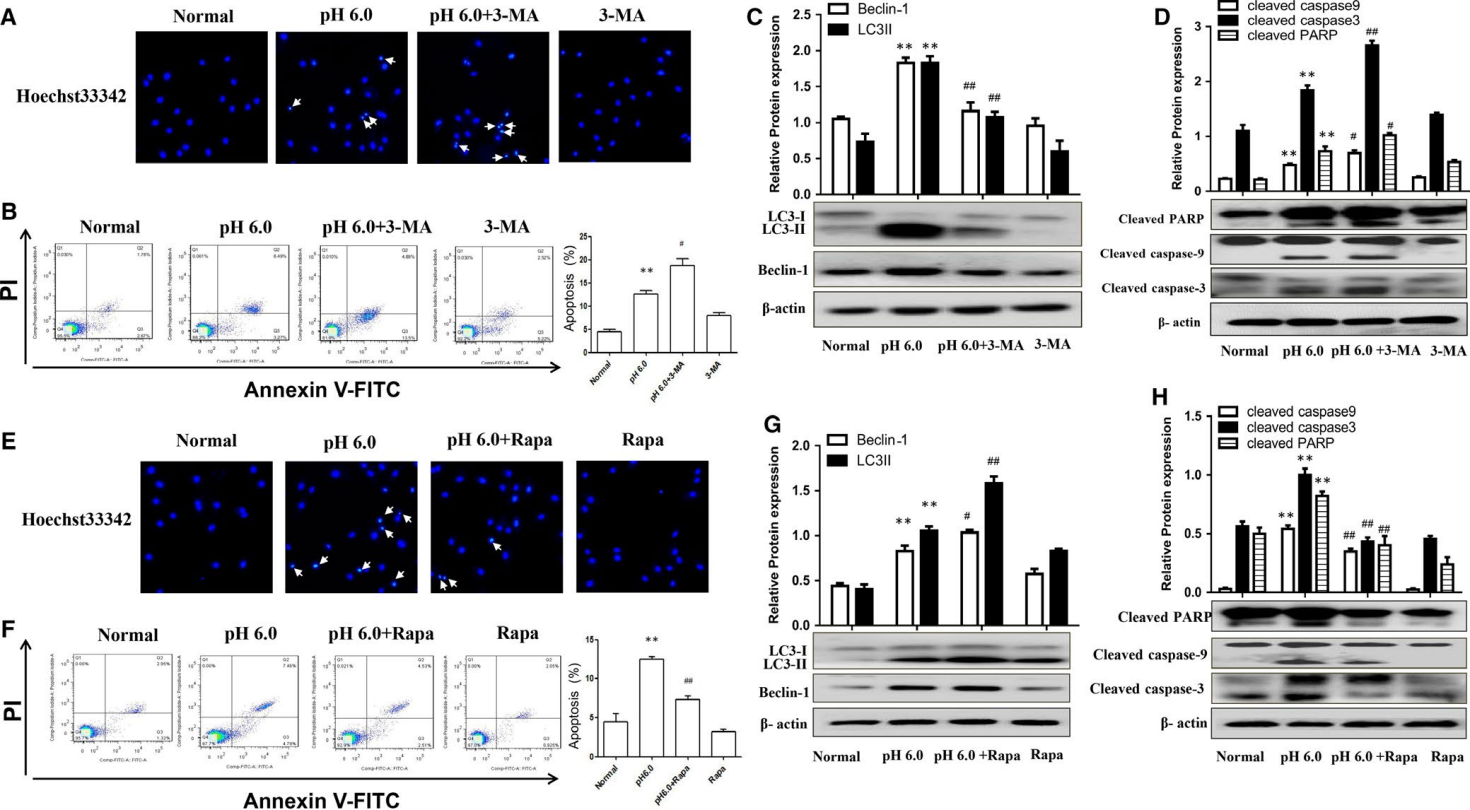
Effects of autophagy on acid‐induced articular chondrocyte apoptosis in vitro. A and E, Hoechst33342 staining for detecting chondrocyte apoptosis. B and F, Chondrocytes apoptosis rates were assessed by flow cytometry. C and G, The LC3‐II and Beclin‐1 expression were detected by Western blotting. D and H, The cleaved PARP, cleaved caspase‐3 and cleaved caspase‐9 expression were assessed by Western blotting. **P < .01 vs normal group; ^#^ *P* < .05, ^##^ *P* < .01 vs pH 6.0 group

We confirmed the effect of autophagy on chondrocyte apoptosis in vitro by inducing rat articular chondrocyte apoptosis by treatment with extracellular acid (pH 6.0), as described previously.^9^ Hoechst 33342 stain showed that chondrocytes undergoing apoptosis were observed in the pH 6.0 group, which could be enhanced by 3‐MA (Figure 2A). Furthermore, the apoptosis rate was increased following acid treatment, and this was also enhanced by 3‐MA (Figure 2B). Extracellular acid (pH 6.0) significantly induced LC3‐II and Beclin‐1 expression in chondrocytes, which effect could be reversed by pre‐treatment with 3‐MA (Figure 2C). Moreover, pH 6.0 treatment also up‐regulated cleaved PARP, cleaved caspase‐9 and cleaved caspase‐3, and this effect was further enhanced by 3‐MA (Figure 2D). These above results suggested that inhibition of autophagy by 3‐MA could enhance acid‐induced apoptosis of rat articular chondrocytes.

### 3.5 Induction of autophagy by Rapa inhibited apoptosis in primary rat articular chondrocytes

We further explored the effect of autophagy activation with Rapa on chondrocyte apoptosis. Hoechst 33342 stain showed that Rapa could reduce the bright blue nuclei and chromatin condensation in acid‐induced chondrocytes (Figure 2E). Flow cytometry results confirmed that Rapa could significantly reduce acid‐induced chondrocyte apoptosis (Figure 2F). LC3‐II and Beclin‐1 in chondrocytes were up‐regulated by pH 6.0 treatment, and this was further enhanced by pre‐treatment with Rapa (Figure 2G). Furthermore, Rapa significantly down‐regulated cleaved PARP, cleaved caspase‐9 and cleaved caspase‐3 in articular chondrocytes (Figure 2H). Taken together, these results indicated that the induction of autophagy by Rapa could reduce articular chondrocyte apoptosis.

## 4 DISCUSSION

Articular chondrocyte death is a major factor leading to reduced capacities of the articular cartilage for repair and regeneration. Multiple death types have been shown to occur in RA articular chondrocytes, among which autophagy and apoptosis have been focuses of attention.^10^ Increasing research has focused on the relationship between autophagy and apoptosis, which contribute to the progression of various rheumatic diseases, including RA.^11^ Although autophagy and apoptosis are two different forms of programmed death, the relationship between them in relation to RA remains unclear. In the current study, we demonstrated that autophagy and apoptosis were alternated dynamically in the pathological process of RA, suggesting that autophagy and apoptosis were involved in the death of articular chondrocytes of RA.

Interestingly, the complex relationships between these processes may appear to be contradictory in different diseases or different conditions. Zhang et al^12^ found that autophagy facilitated apoptosis in human lung cancer cells treated with Paris Saponin II, while Song et al^13^ showed that apoptosis and autophagy antagonized each other, such that apoptosis could be inhibited by autophagy, thus promoting cell survival.^14^ Our results showed that treatment with the autophagy inhibitor 3‐MA increased joint inflammation and articular cartilage injury in AA rats, while the autophagy activator Rapa reduced synovial fluid inflammation and articular chondrocyte apoptosis in vivo. In addition, pre‐treatment with 3‐MA enhanced acid‐induced apoptosis of rat articular chondrocytes, while pre‐treatment with Rapa inhibited this process in vitro. These results indicated that autophagy activation could ameliorates AA rat articular chondrocyte injury by inhibiting apoptosis, suggesting that autophagy might be a potential target for the treatment of RA.

Evidence suggests that autophagy protects meniscal cells from glucocorticoid‐induced apoptosis via inositol trisphosphate receptor signalling.^15^ Furthermore, regarding chondrocytes, autophagy self‐activation acts a protective mechanism against apoptosis under short‐term glucocorticoid treatment.^16^ The present study also provided evidence for autophagy have the protective effect on AA rat articular cartilage via inhibiting apoptosis, we just investigated the effects of autophagy on apoptosis of articular chondrocytes in AA rats, and more studies are needed to research the potential molecular mechanism between autophagy and apoptosis.

In summary, our results indicated that autophagy plays a protective role in articular chondrocytes during the pathogenesis of RA via suppressing chondrocyte apoptosis. We provide the first evidence of dynamic changes in autophagy and apoptosis of articular chondrocytes in AA rats and of the important roles of autophagy in modulating chondrocyte apoptosis. These results suggest that autophagy might be a potential target for the treatment of RA.

## CONFLICT OF INTEREST

The authors declare no conflict of interest.

## **AUTHOR** **CONTRIBUTIONS**

RZ, FZ and XW: performed the experiments, analysed the data; WH and FC: designed experiments; RZ and FZ: wrote the manuscript; YC, CZ, SS and XQ: helped to perform the experiments and prepare the materials; BD and KW designed the study's analytic strategy. All authors read and approved the manuscript.

## Data Availability

The data that support the findings of this study are available from the corresponding author upon reasonable request.
